# Impact of frailty on 5-year survival in patients older than 70 years undergoing colorectal surgery for cancer

**DOI:** 10.1186/s12957-021-02221-6

**Published:** 2021-04-10

**Authors:** Manuel Artiles-Armas, Cristina Roque-Castellano, Roberto Fariña-Castro, Alicia Conde-Martel, María Asunción Acosta-Mérida, Joaquín Marchena-Gómez

**Affiliations:** 1 Department of General Surgery, Hospital Universitario de Gran Canaria Doctor Negrín, Barranco La Ballena s/n, 35012 Las Palmas de Gran Canaria, Spain; 2 Universidad de Las Palmas de Gran Canaria, Las Palmas de Gran Canaria, Spain; 3 Department of Anaesthesiology, Hospital Universitario de Gran Canaria Doctor Negrín, Barranco La Ballena s/n, 35012 Las Palmas de Gran Canaria, Spain; 4 Department of Internal Medicine, Hospital Universitario de Gran Canaria Doctor Negrín, Barranco La Ballena s/n, 35012 Las Palmas de Gran Canaria, Spain

**Keywords:** Frailty, Geriatrics, Colorectal cancer, Comorbidity

## Abstract

**Background:**

Frailty has been shown to be a good predictor of post-operative complications and death in patients undergoing gastrointestinal surgery. The aim of this study was to analyze the differences between frail and non-frail patients undergoing colorectal cancer surgery, as well as the impact of frailty on long-term survival in these patients.

**Methods:**

A cohort of 149 patients aged 70 years and older who underwent elective surgery for colorectal cancer was followed-up for at least 5 years. The sample was divided into two groups: frail and non-frail patients. The Canadian Study of Health and Aging-Clinical Frailty Scale (CSHA-CFS) was used to detect frailty. The two groups were compared with regard to demographic data, comorbidities, functional and cognitive statuses, surgical risk, surgical variables, tumor extent, and post-operative outcomes, which were mortality at 30 days, 90 days, and 1 year after the procedure. Univariate and multivariate analyses were also performed to determine which of the predictive variables were related to 5-year survival.

**Results:**

Out of the 149 patients, 96 (64.4%) were men and 53 (35.6%) were women, with a median age of 75 years (IQR 72–80). According to the CSHA-CFS scale, 59 (39.6%) patients were frail, and 90 (60.4%) patients were not frail. Frail patients were significantly older and had more impaired cognitive status, worse functional status, more comorbidities, more operative mortality, and more serious complications than non-frail patients. Comorbidities, as measured by the Charlson Comorbidity Index (*p* = 0.001); the Lawton-Brody Index (*p* = 0.011); failure to perform an anastomosis (*p* = 0.024); nodal involvement (*p* = 0.005); distant metastases (*p* < 0.001); high TNM stage (*p* = 0.004); and anastomosis dehiscence (*p* = 0.013) were significant univariate predictors of a poor prognosis on univariate analysis. Multivariate analysis of long-term survival, with adjustment for age, frailty, comorbidities and TNM stage, showed that comorbidities (*p* = 0.002; HR 1.30; 95% CI 1.10–1.54) and TNM stage (*p* = 0.014; HR 2.06; 95% CI 1.16–3.67) were the only independent risk factors for survival at 5 years.

**Conclusions:**

Frailty is associated with poor short-term post-operative outcomes, but it does not seem to affect long-term survival in older patients with colorectal cancer. Instead, comorbidities and tumor stage are good predictors of long-term survival.

## Introduction

An ageing population is increasing the demand for healthcare. More than 4 million major surgical operations are performed annually in the USA on older patients, yet as an increasing number of geriatric patients undergo surgery, there is a clear increase in age-related peri-operative morbidity and mortality [1]. Many of these operations are surgical procedures to treat older patients with colorectal cancer (CRC). In fact, colorectal cancer is the third most common cancer in the world, and surgery, with either curative or palliative intent, is the main treatment modality for this disease. Approximately 60% of CRC patients are > 70 years old at the time of diagnosis, and 43% are > 75 years of age [2].

On the other hand, the pre-operative detection of frailty is becoming more relevant in these older surgical patients. Frailty has been shown to be a good predictor of post-operative complications of major gastrointestinal procedures [3], and it has been associated with post-operative mortality across all non-cardiac surgical specialties [4]. Additionally, frailty has a detrimental impact on costs and hospital profit for elective surgery [5]. Many reports suggest that frailty screening should be included in pre-operative assessments to enhance surgical decision-making and patient counseling [6–9].

In a systematic review regarding frailty in CRC surgical patients, Fagard et al. [10], found that only five quality articles with small numbers of patients and various definitions of frailty and post-operative outcomes, which made comparisons difficult. Recently, additional studies have been reported involving frail patients operated on for CRC, either in the elective setting [11, 12] or in the emergency setting [13], including a meta-analysis [14]. They also found that frailty is a robust predictor of severe post-operative complications in patients with colorectal cancer. However, the differences in long-term outcomes between frail and non-frail patients operated on for colorectal cancer have been less well documented. Furthermore, when assessing long-term results, in most of these studies, there is no adjustment for possible confounding factors related to the evolution of a neoplasm, such as tumor stage.

The aim of this study was to analyze the pre-, intra-, and post-operative differences in characteristics between older frail and non-frail patients with CRC and to investigate the long-term prognosis of these patients after adjusting for frailty, comorbidities, and tumor stage.

## Methodology

### Study design and participants

An observational study was conducted in a cohort of 149 consecutive patients older than 70 years old who underwent elective colorectal surgery for cancer between January 2013 and December 2015. Data were collected prospectively by a single surgeon and recorded in a database. The setting was a tertiary hospital that is responsible for a population of approximately 400,000 people. The study was approved by the Ethics Committee of the hospital (Code 140195). All patients consented to participate in the study.

### Method

A surgeon and an anaesthesiologist pre-operatively evaluated all patients, and a complete anamnesis and physical examination were completed. The pre-operative geriatric assessment was performed by a specifically trained surgeon (MAA), regardless of the surgeon who operated on the patient. The geriatric evaluation usually lasted half an hour. The diagnosis of CRC was made by colonoscopy and biopsy. All patients underwent preoperative thoraco-abdominal tomography to determine the extent of disease. Laboratory tests, electrocardiograms, and additional tests were also performed based on each patient’s underlying condition. The anaesthesiologist did not normally refuse to administer anesthesia if the surgeon and family had agreed to undergo the procedure despite the presence of comorbidities or disabilities that were possible contraindications. In fact, there were no patients rejected for surgery, neither did any patient refuse surgery.

All the surgical procedures were performed by a staff surgeon, and reconstruction of the transit after resection was usually performed by mechanical anastomosis.

The cohort was divided into two groups: frail patients and non-frail patients.

The Clinical Frailty Score from the Canadian Study of Health and Aging (CSHA-CFS) was used to evaluate frailty in each patient. This instrument, which was proposed by Rockwood et al. [15], is based on a numerical scale from 1 to 7 as follows: CFS 1 (very fit), CFS 2 (well), CFS 3 (well with treated comorbid disease), CFS 4 (apparently vulnerable), CFS 5 (mildly frail), CFS 6 (moderately frail), and CFS 7 (severely frail). We considered CSHA-CFS ≥ 4 as a threshold for determining frailty as it has been recently suggested that this cut-off highly correlates with postoperative outcomes [11].

The two groups were compared with regard to demographic data, comorbidities, functional and cognitive statuses, surgical risk, surgical variables, tumor extent, and post-operative outcomes, which were mortality at 30 days, 90 days, and 1 year after the procedure. All patients were followed for 5 years. Therefore, survival at 5 years was also recorded. No patients were lost to follow-up.

The following variables were evaluated:

#### Patient characteristics

Age and sex were recorded. Regarding the age cut-off point, the progressive increase in life expectancy in Western countries led us to consider it appropriate to include patients aged ≥70, which is 5 years older than the World Health Organization definition of the older population.

#### Preoperative status

##### Charlson Comorbidity Index (ChCI)

The ChCI score was calculated pre-operatively for each patient. This score includes 19 medical conditions with assigned point values of 1, 2, 3, or 6, with totals ranging from 0 to 37 points. The absence of comorbidity is represented by 0 points; low levels of comorbidity are 1–2 points; moderate levels of comorbidity are 3–4 points; and high levels of comorbidity are > 4 points [16]. In this study, the ChCI was not adjusted for age or for the prevalence of AIDS [17], as there were no cases of this in the study population.

##### ASA (American Society of Anesthesiology) physical status classification system

This scale was developed to offer clinicians a simple categorization of a patient’s physiological status that could be helpful in predicting operative risk [18].

##### Functional status

The functional status with regard to the basic activities of daily living (ADL) was determined using the Barthel Index [19]. The total score for this index ranges from 0, corresponding to a total dependence, to 100 points, corresponding to complete independence. For analytical purposes, this variable was categorized as independent (80–100 points) versus some grade of dependency (< 80 points) [20].

The previous functional status with regard to the Instrumental Activities of Daily Living (IADL) was also evaluated using the Lawton-Brody Index [21]. In summary, the score ranges from 0 (low function, dependent) to 8 points (high function, independent) for women and from 0 to 5 for men.

##### Cognitive status

The Short Portable Mental State Questionnaire (SPMSQ) with the Pfeiffer test [22] was performed. This short questionnaire (10 items) provides an estimate of a patient’s cognitive status according to the number of incorrect answers to basic questions, with values ranking from 0–1 (no impairment) to 9–10 (most severe impairment). In this study, the cut-off value was arbitrarily set at < 3 versus ≥ 3 errors.

##### Body Mass Index and Mini Nutritional Assessment Short Form questionnaire (MNA-SF) [23]

The MNA-SF is a 6-item assessment tool based on the patient’s body mass index (BMI), a dietary questionnaire and a subjective assessment. The maximum score is 14 points; the risk of malnutrition increases with decreasing scores.

##### Laboratory values

The values of hemoglobin (gr/dL), serum creatinine (mg/dL), and serum albumin (gr/dL) were recorded.

##### Surgical variables

The surgical variables were the type of surgical procedure performed, the use of a laparoscopic approach, the generation of an anastomosis (no/yes), and the need for at least one red blood cell unit transfusion during and/or immediately before or after the procedure (48 h).

##### Cancer stage (TNM)

Tumor stage was recorded according to the 8th edition of the American Joint Committee on Cancer staging system and was categorized as stage I–II vs stage III–IV.

##### Post-operative complications

Post-operative complications were graded using the Comprehensive Complication Index (CCI) [24]. This score summarizes all post-operative complications and seems to be more sensitive than other existing scales. The values of the index range from 0 (uneventful course) to 100 points (death). The Clavien-Dindo classification [25] was also used to assess the severity of post-operative complications. This variable was categorized into two categories: minor complications (grades I–II) and major complications (grades III–V).

##### Hospital stay

The post-operative hospital stay of each patient was collected and registered.

##### Mortality

Post-operative mortality, defined as any death within 30 days after the surgical procedure, 90-day mortality, and 1-year mortality after surgery, was also recorded.

##### Long-term survival

All patients were followed for at least 5 years or until death. Their status was monitored through their medical history or telephone contact with either the patients themselves or their relatives. Long-term survival was considered as the period between the performance of the surgical procedure and death or the date of the last follow-up observation before the analysis, if the subject was still alive. The mean follow-up duration in the cohort was 5 years.

### Statistical analysis

The data were analyzed using the statistical package SPSS 26.0 for Windows (IBM Corporation, Armonk, NY, USA). Categorical variables are summarized as frequencies and percentages; continuous variables are described as the means and standard deviations (SD) when the data followed a normal distribution or as medians and interquartile ranges (IQRs) when they did not. The Kolmogorov-Smirnov test was applied to evaluate the normality of the distribution of values in continuous variables.

Univariate analysis was performed to compare the characteristics of non-frail and frail patients with regard to pre-operative features, surgical variables, tumor extent, and post-operative outcomes.

The chi-squared test or Fisher’s test was used to compare categorical data. For parametric distributions, Student’s *t* test was used to compare the mean values of the two groups. For ordinal variables or non-parametric variables, the Mann–Whitney *U* test was used to compare the median values of the response variable.

Likewise, another univariate analysis was performed to compare the survival curves based on different independent variables. The survival curves were constructed using the Kaplan-Meier method. The log-rank test was applied to compare survival at 5 years.

Finally, multivariate Cox proportional hazards regression analysis was conducted. The primary purpose of the multivariate analysis was to adjust the variables habitually related to long-term survival (age, comorbidities, tumor stage) by the variable frailty, regardless of whether those variables were significant or not in the univariate analysis. Multicollinearity was tested using the variance inflation factor (VIF).

Statistical significance was defined as *p* < 0.05. The hazard ratio (HR) and 95% confidence interval (95% CI) were also calculated as measurements of associations using Cox regression.

## Results

Out of the 149 patients, 96 (64.4%) were men and 53 (35.6%) were women, with a median age of 75 years (IQR 72–80). Only one patient was institutionalized. The rest of the patients lived at home with at least one relative and/or a caregiver.

According to the CSHA-CFS scale, 86 (57.7%) patients were grades 1–3; 44 (29.5%) patients were grades 4–5, and 19 (22.7%) were grades 6–7. After categorizing the variable (< 4 versus ≥ 4), 59 (39.6%), patients were considered frail, and 90 (60.4%) patients were not frail.

### Pre-operative status

Forty-seven patients (31.5%) were classified as ASA I–II, and 102 (68.5%) were classified as ASA III–IV. The median ChCI score was 3.0 (IQR 2.0–4.0). Fourteen patients (9.4%) had a Barthel Index score < 80 points, and 135 (90.6%) had a Barthel Index score ≥ 80 points. The median value of the Lawton-Brody Index score was 6.0 (IQR 5.0–8.0). According to the Pfeiffer test, 140 (94%) patients had normal mental functioning, and 9 (6%) patients had cognitive impairment.

The mean body mass index was 26.8 kg/m^2^ (SD ± 26.8). The median value of the MNA-SF test was 10.0 (IQR 9.0–12.0).

The mean level of hemoglobin was 12.5 g/dL (SD ± 2.2), the median level of serum creatinine was 0.96 mg/dL (IQR 0.79–1.13), and the mean level of serum albumin was 3.8 g/dL (SD ± 0.5).

### Surgical variables

The following procedures were performed: right colectomy (70 patients), transverse colectomy (1 patient), left colectomy (12 patients), sigmoidectomy (17 patients), rectal anterior resection (33 patients), Hartmann procedure (3 patients), subtotal colectomy (3 patients), total colectomy (1 patient), abdominoperineal resection (8 patients), and resection of pelvic recurrence of rectal cancer (1 patient).

The laparoscopic approach was performed in 56 (38.9%) procedures, and anastomosis was carried out in 127 (85.2%) patients.

Peri-operatively, 33 (22.1%) patients received at least one red blood transfusion.

### Tumor extent

In 45 (30.2%) patients, the tumor did not extend past the muscularis propria layer (T1–T2), and in 104 (69.8%) patients, the tumor invaded through the muscularis propria into peri-colorectal tissues or penetrated the visceral peritoneum or other organs (T3–T4). Likewise, 102 (68.5%) patients did not have lymph node involvement (N0), and 47 (31.5%) had lymph node involvement (N1). Only 7 (4.7%) patients had distant metastasis (M1).

According to the 8th edition of the American Joint Committee on Cancer Staging, 99 (66.4%) patients were classified as having TNM stage I–II disease, and 50 (33.6%) patients were classified as having stage III–IV disease.

Of the 7 patients with distant metastases at diagnosis, only 2 patients underwent curative surgery (liver metastasectomy). In the other 5 patients, resection of the primary tumor was performed on a palliative basis because the neoplasm was highly symptomatic.

### Post-operative complications

Seventy-four patients (49.7%) had at least 1 post-operative complication, of whom 42 (28.25%) patients were classified as Clavien–Dindo grades I–II (minor complications), and 32 (21.5%) patients grades III–V (major complications), including 5 deaths. The median Comprehensive Complication Index score was only 8.7 (IQR 0.0–24.2). Within the group of patients who had complications (CCI ≥ 1), the median CCI score was 33.3 (IQR 8.7–46.3). Anastomosis dehiscence was observed in 10 patients (7.9% of the patients with anastomosis).

### Outcomes

The median post-operative hospital stay was 10 days (IQR 7–15). Hospital stay was associated with the severity of complications (*p* < 0.001). The median hospital stay of the patients without complications was 7 days (IQR 6.0–9.0), while the median hospital stay of the patients with minor complications was 13 days (IQR 9.0–16.0), and the median hospital stay of the patients with major complications was 26 days (IQR 15.0–38.5).

The operative mortality rate (30 days) was 3.4% (5 patients). The causes of death were anastomotic dehiscence (2 patients), cardiogenic shock (1 patient), pneumonia (1 patient), and venous mesenteric ischemia due to massive venous thrombosis (1 patient).

The 90-day mortality rate was 8.1% (12 patients), and the 1-year mortality rate was 12.8% (19 patients).

By the end of the follow-up period, 48 (17.6%) patients had died. The cumulative survival rates at 3 and 5 years were 78.4% and 68%, respectively. Out of the 43 patients who died during follow-up, 21 (48.8%) patients died due to tumor progression, and 22 (51.2%) patients died due to non-tumor-related causes.

Regarding chemotherapy, only 37 (24.8%) patients received neo- or adjuvant chemotherapy. In 10 (6.7%) cases, it was administered as neoadjuvant therapy. The patients in whom chemotherapy was not administered were mainly due to comorbidity or an advanced degree of frailty.

**Fig. 1:**
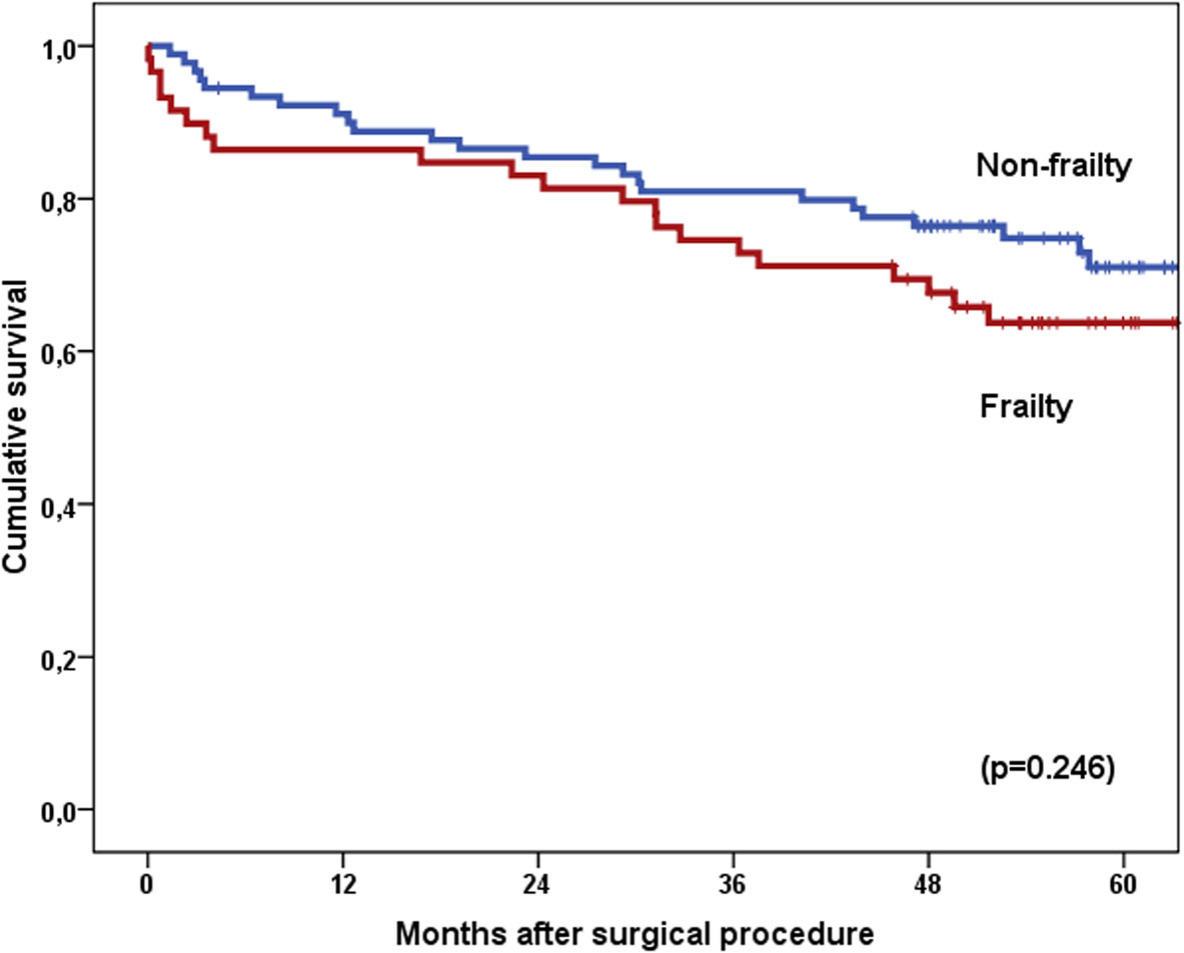
Differences in cumulative survival between non-frail and frail patients. Log-rank test (*p* = 0.246)

The results of the comparisons between frail and non-frail patients are summarized in Table 1. Frail patients were significantly older, were more likely to have impaired cognition, and had a worse functional status, more comorbidities, a higher operative mortality rate, and more serious complications than non-frail patients. However, there were no significant differences in mortality between these two groups at 90 days and 1 year after the surgical procedure. Furthermore, although a smaller proportion of the frail patients than the non-frail patients were alive at 5 years, the survival analysis did not show statistically significant difference between the two groups. The mean survival time in frail patients was 58.9 months, whereas non-frail patients had a mean survival of 63.9 months (*p* = 0.246) (Fig. 1).

**Table 1:** Comparative analysis between frail and non-frail patients with CRC, according to the CSHA-CSF scale

Variable	Total *N* (%) 149 (100)	No frailty *N* (%) 90 (60.4%)	Frailty *N* (%) 59 (39.6%)	*P*
Age				
Median (IQR)	75 (72–80)	74 (72–29)	77 (73–81)	0.008*
Gender				
Men	96 (64.4)	61 (67.8)	35 (59.3)	0.292
Women	53 (35.6)	29 (32.2)	24 (40.7)	
ASA				
I–II	47 (31.5)	32 (35.6)	15 (25.4)	0.193
III–IV	102 (68.5)	58 (64.4)	44 (74.6)	
Charlson Index				
Median (IQR)	3.0 (2.0–4.0)	2.0 (2.0–4.0)	3.0 (2.0–4.0)	0.005*
Barthel				
< 80	14 (9.4)	1 (1.1)	13 (22.0)	< 0.001*
≥ 80	135 (90.6)	89 (98.9)	46 (78.0)	
Lawton-Brody				
Median (IQR)	6.0 (5.0–8.0)	7.0 (6.0–8.0)	4.0 (3.0–6.0)	< 0.001*
Pfeiffer				
< 3	140 (94.0)	89 (98.9)	51 (86.4)	0.003*
≥ 3	9 (6.0)	1 (1.1)	8 (13.6)	
BMI^a^				
Mean ± SD	26.8 (± 4.0)	26.9 (± 4.2)	26.5 (± 3.7)	0.746
MNA^b^				
Median–IQR	10.0 (9.0–12.0)	11.0 (9.0–13.0)	10.0 (9.0–12.0)	0.185
Hemoglobin gr/dL				
Mean (±SD)	12.5 (± 2.2)	12.6 (± 2.1)	12.3 (± 2.2)	0.507
Creatinine mg/dL				
Median (IQR)	0.96 (0.79–1.13)	0.96 (0.82–1.06)	0.97 (0.75–1.22)	0.840
Albumin gr/dL				
Mean (±SD)	3.8 (± 0.5)	3.8 (± 0.5)	3.8 (± 0.5)	0.963
Laparoscopic approach				
*n* (%)	56 (38.9)	30 (53.6)	26 (46.4)	0.230
Anastomosis				
No	22 (14.8)	13 (14.4)	9 (15.3)	0.892
Yes	127 (85.2)	77 (85.6)	50 (84.7)	
Transfusions				
No	116 (77.9)	71 (78.9)	45 (76.3)	0.707
Yes	33 (22.1)	19 (21.1)	14 (23.7)	
T				
1–2	45 (30.2)	25 (27.8)	20 (33.9)	0.426
3–4	104 (69.8)	65 (72.2)	39 (66.1)	
N				
0	102 (68.5)	60 (66.7)	42 (71.2)	0.561
1	47 (31.5)	30 (33.3)	17 (28.8)	
M				
0	142 (95.3)	87 (96.7)	55 (93.2)	0.436
1	7 (4.7)	3 (3.3)	4 (6.8)	
TNM stage				
I–II	99 (66.4)	59 (65.6)	40 (67.8)	0.777
III–IV	50 (33.6)	31 (34.4)	19 (32.2)	
Anastomosis dehiscence				
No	139 (93.3)	86 (95.6)	53 (89.8)	0.195
Yes	10 (6.7)	4 (4.4)	6 (10.2)	
CCI ≥ 1^c^				
Median (IQR)	33.3 (8.7–46.3)	21.8 (8.7–41.3)	32.4 (20.9–55.4)	0.04*
Hospital stay				
Median (IQR)	10 (7–15)	10 (7–16)	9 (7–15)	0.259
Chemotherapy				
No	112 (75.2)	60 (66.7)	52 (88.1)	0.003*
Yes	37 (24.8)	30 (33.3)	7 (11.9)	
30-day mortality				
No	144 (96.6)	90 (100.0)	54 (91.5)	0.009*
Yes	5 (3.4%)	0 (0.0)	5 (8.5)	
90-day mortality				
No	137 (91.9)	85 (94.4)	52 (88.1)	0.166
Yes	12 (8.1)	5 (5.6)	7 (11.9)	
1-year mortality				
No	130 (87.2)	80 (88.9)	50 (84.7)	0.458
Yes	19 (12.8)	10 (11.1)	9 (15.3)	
Cumulative survival at 5 years				
(mean months)	62.16	63.9	58.9	0.246
Death from non-tumoral causes				
*n* (%)	21 (43.8)	11 (42.3)	16 (72.7)	0.034*

^a^*BMI* body mass index

^b^*MNA* Mini-Nutritional-Assessment

^c^*CCI* Comprehensive complication index

*Statistically significant

Univariate analyses of the factors related to long-term survival are summarized in Table 2. Comorbidities, as measured by the Charlson Comorbidity Index (*p* = 0.001); the Lawton-Brody Index (*p* = 0.011); failure to perform an anastomosis (*p* = 0.024); nodal involvement (*p* = 0.005); distant metastases (*p* < 0.001); high TNM stage (*p* = 0.004); and anastomosis dehiscence (*p* = 0.013) were significant univariate predictors of a poor prognosis.

**Table 2:** Univariate analysis of long-term survival using Cox regression for each variable

Variables	Total ***N*** (%) 149 (100)	Alive 101 (67.8%)	Death 48 (32.2%)	***P***	HR (CI 95%)
Age					
Median (IQR)	75 (72–80)	74 (72–79)	78 (73–80.75)	0.140	1.03 (0.99–1.09)
Gender					
Men	96 (64.4)	61 (60.4)	35 (72.9)	0.074	0.56 (0.30–1.07)
Women	53 (35.6)	40 (39.6)	13 (27.1)		
ASA					
I–II	47 (31.5)	36 (35.6)	11 (22.9)	0.241	1.50 (0.76–2.94)
III–IV	102 (68.5)	65 (64.4)	37 (77.1)		
Charlson Index					
Median (IQR)	3.0 (2.0–4.0)	3.0 (2.0–3.0)	3.0 (2.0–5.0)	0.001*	1.37 (1.17–1.60)
Barthel					
< 80	14 (9.4)	8 (7.9)	6 (12.5)	0.314	0.64 (0.27–1.52)
≥ 80	135 (90.6)	93 (92.1)	42 (87.5)		
Lawton-Brody					
Median (IQR)	6.0 (5.0–8.0)	7.0 (5.0–8.0)	6.0 (4.0–7.0)	0.011*	0.85 (0.76–0.97)
Pfeiffer					
< 3	140 (94.0)	97 (96.0)	43 (89.6)	0.157	1.95 (0.77–4.94)
≥ 3	9 (6.0)	4 (4.0)	5 (55.6)		
BMI^a^					
Mean ± SD	26.8 (± 4.0)	26.7 (± 4.1)	26.9 (± 3.7)	0.938	1.00 (0.94–1.07)
MNA^b^					
Median IQR	10.0 (9.0–12.0)	10.0 (9.0–12.5)	10.5 (9.0–12.0	0.588	0.97 (0.85–1.09)
Frailty					
No	90(60.4)	64 (63.4)	26 (54.2)	0.249	1.40 (0.79–2.47)
Yes	59 (39.6)	37 (36.6)	22 (45.8)		
Hemoglobin gr/dL					
Mean (±SD)	12.5 (± 2.2)	12.6 (± 2.1)	12.3 (± 2.3)	0.703	0.98 (0.86–1.11)
Creatinine mg/dL					
Median (IQR)	0.96 (0.79–1.13)	0.94 (0.79–1.07)	1.00 (0.80–1.33)	0.060	1.66 (0.98–2.81)
Albumin gr/dL					
Mean (±SD)	3.8 (± 0.5)	3.8 (± 0.5)	3.7 (± 0.5)	0.430	0.749 (0.37–1.54)
Laparoscopic approach					
*n* (%)	56 (38.9)	41 (40.6)	15 (31.3)	0.118	0.635 (0.36–1.12)
Anastomosis					
No	22 (14.8)	10 (9.9)	12 (25.0)	0.024*	0.47 (0.24–0.91)
Yes	127 (85.2)	91 (90.1)	36 (75.0)		
Transfusions					
No	116 (77.9)	80 (79.2)	36 (75.0)	0.516	1.24 (0.65–2.39)
Yes	33 (22.1)	21 (20.8)	12 (25.0)		
T					
1–2	45 (30.2)	34 (33.7)	11 (22.9)	0.228	1.51 (0.77–2.97)
3–4	104 (69.8)	67 (66.3)	37 (77.1)		
N					
0	102 (68.5)	76 (75.2)	26 (25.5)	0.005*	2.27 (1.29–4.01)
1	47 (31.5)	25 (24.8)	22 (45.8)		
M					
0	142 (95.3)	100 (99.0)	42 (87.5)	< 0.001*	6.21 (2.59–14.93)
1	7 (4.7)	1 (1.0)	6 (12.5)		
TNM stage					
I–II	99 (66.4)	74 (73.3)	25 (52.1)	0.004*	2.29 (1.30–4.04)
III–IV	50 (33.6)	27 (26.7)	23 (15.4)		
Anastomosis dehiscence					
No	139 (93.3)	97 (96.0)	42 (87.5)	0.013*	2.95 (1.25–6.96)
Yes	10 (6.7)	4 (4.0)	6 (12.5)		
Chemotherapy					
No	112 (75.2)	75 (74.3)	37 (77.1)	0.890	0.95 (0.49–1.87)
Yes	37 (24.8)	26 (25.7)	11 (22.9)		

^a^*BMI* body mass index

^b^*MNA* Mini-Nutritional-Assessment

*Statistically significant

Multivariate analysis of long-term survival, with adjustment for age, frailty, comorbidities, and TNM stage, showed that comorbidities (*p* = 0.002; HR 1.30; 95% CI 1.10–1.54) and TNM stage (*p* = 0.014; HR 2.06–95% CI 1.16–3.67) were the only independent risk factors for survival at 5 years (Table 3). No multicollinearity was detected among the independent variables.

**Table 3:** Multivariate analysis (Cox regression) of long-term survival, adjusting for age, frailty, comorbidity, and TNM stage

Variables	***B***	SE	Wald	***p***	HR (95.0% CI)
Age	0.028	0.026	1.229	0.268	1.03 (0.98–1.08)
TNM stage	0.723	0.295	5.999	0.014*	2.06 (1.16–3.67)
Charlson Comorbidity Index	0.263	0.085	9.496	0.002*	1.30 (1.10–1.54)
Frailty	0.044	0.315	0.019	0.889	1.05 (0.56–1.94)

*Statistically significant. *B* regression coefficient, *CI* confidence interval, *HR* hazard ratio, *SE* standard error, *Wald* test statistic

## Discussion

This study showed that frail patients were significantly older, were more likely to have impaired cognition, and had a worse functional status, more comorbidities, a higher operative mortality rate, and more serious complications than non-frail patients. These findings, which are related to early outcomes, are in line with what has recently been published in relation to pre-operative frailty [10].

In recent years, there has been an emphasis on the fact that a lack of adequate physiological reserves affects the survival of older patients undergoing surgical procedures. Frailty has been defined as a multifactorial syndrome characterized by decreased reserves and less resistance to stressors, resulting from a cumulative decline across multiple physiological systems and the subsequent vulnerability to adverse outcomes [26]. This concept was previously applied, in general, only to non-surgical patients, and there is still no clear consensus regarding its application to elderly surgical patients [27]. Nonetheless, frailty has become an emerging risk stratification measure in surgical risk patients and may also be a valuable quality metric [12].

Therefore, for many authors, an assessment of frailty is essential for estimating the overall and functional outcomes in geriatric surgical patients, depending on the planned intervention [28].

For this purpose, the pre-operative performance of the process called the comprehensive geriatric assessment (CGA) is recommended. Ellis et al. [29] defined CGA as a multidimensional diagnostic and therapeutic process that is focused on determining a frail older person’s medical, functional, mental, and social capabilities and limitations with the goal of ensuring that problems are identified, quantified, and managed appropriately. The International Society of Geriatric Oncology has recommended the use of the CGA to guide the development of an oncologic treatment plan in older patients with cancer, including those who need to undergo surgery [30]. Nevertheless, there is also a current trend to use previously defined and highly useful frailty scales to detect this deficiency, such as the CSHA-CFS score [15] or the different versions of the Modified Frailty Index of the American College of Surgeons National Surgical Quality Improvement Program (ACS-NSQIP) [12, 31].

Focusing on colorectal cancer surgery, two systematic reviews [10, 14] also reported the same conclusions: frailty is a good predictor of post-operative complications after elective colorectal surgery. Therefore, assessing frailty in colorectal oncology seems important to determining the operative risks and benefits and to guiding peri-operative management. However, the relationship between frailty and long-term survival has not been well studied [10, 31]. Most studies report 30-day mortality [13, 14, 31–34], 3-month mortality [35], and 1-year mortality as output variables [35]. Few studies [36] have provided a follow-up of this population at 5 years. Furthermore, the variable “frailty” in these reports is not usually adjusted for possible confounders such as age, comorbidities and tumor stage. Only Ommundsen et al. [36] reported the results of a multivariable analysis adjusting frailty for TNM stage, age, and sex in older patients operated on for CRC; however, there was no adjustment for comorbidities. These authors studied 1-year and 5-year survival rates in this population. The comparison between frail and non-frail older patients showed survival rates of 80% and 92%, respectively, for 1-year survival and 24% and 66%, respectively, for 5-year survival. They concluded that the impact of frailty on 5-year survival is comparable with that of TNM stage after CRC surgery. These results differ from those obtained in our series. We observed that the long-term survival of frail patients operated on for colorectal cancer was fundamentally related to comorbidities and tumor stage. Therefore, although operative mortality is higher in frail patients than in non-frail patients, frailty per se does not seem to be a determining factor for the long-term survival of these patients, even after adjustment for comorbidities and tumor stage. Only one study [35] reached the same conclusions, but that study included a small number of patients and a follow-up period of only 1 year.

The observed differences could be explained if we consider three points of discussion.

First, the definition of the concept of frailty and the method used to assess frailty were different. Although the published literature includes several scales for defining frailty in surgical patients, there is no single gold standard measure for frailty in this context. Multiple frailty screening tools have been developed [8, 37], and their usefulness is somewhat variable among different patient populations, indications for surgery, and surgical procedures performed. The overwhelming number of risk scales developed, most of which have been applied to small populations, has led to few being used consistently in clinical practice.

In our series, the CSHA-CFS was used to determine frailty. It is simple to administer and correlates well with the frailty index, which has been shown to predict morbidity and mortality in some surgical populations [38]. Although this study did not aim to compare the CSHA-CFS with other frailty scales, the CSHA-CFS has certain advantages, such as being less time-consuming, having been validated, and being easy to perform [38]; in addition, it has very good inter-rater reliability [39]. The proportion of patients with frailty in our study was 40%, which is comparable to the proportions reported in the previously published literature (25–46%) [27].

The ACS-NSQIP 11-item Modified Frailty Index (11-mFI) [12], the ACS-NSQIP 5-item Modified Frailty Index (5-mFI) [13, 31], both based on the CSHA scale; the Fried criteria [26, 33]; the Groningen Frailty Indicator [34, 40]; and a series of cut-offs for the components of the pre-operative geriatric assessment [35, 36], have been used to detect frailty by other authors.

Therefore, given the large number of scales used, it is difficult to make comparisons between the series analyzed.

Second, there was confusion between frailty and comorbidities in some of the previously described frailty rating scales. The components of the pre-operative geriatric assessment with cut-off values for frailty used by some authors [35, 36], the 11-mFI [12] and 5-mFI [13, 31] scores mix up, in the same scale, comorbidities with other values used to define frailty. Actually, the terms “frailty,” “disability,” and “comorbidity” may be considered somewhat confusing concepts in older surgical patients. According to Richard et al. [41], there is an overlap of these concepts that may determine the systematic evaluation of the three concepts in all patients. Specifically, frailty and comorbidities are prevalent in older adults and are strongly interrelated. Previously, comorbidities were even considered to be a component of frailty [14]. However, we agree with Fried et al. [26] that frailty may have a biologic basis and be a distinct clinical syndrome. We believe that it is important to distinguish comorbidities from frailty, and it might be appropriate to assess them separately. A patient may have comorbidities and may not be considered frail, and a frail patient may not necessarily have comorbidities. To avoid this bias, in our study, we used the CSHA to define frailty and analyzed comorbidities and disability independently.

Third, the heterogeneity of the studied sample is an important consideration. We included in our series only patients undergoing elective surgery for colorectal cancer. However, other reported series [12, 31] have included patients who underwent any elective or non-elective colorectal procedures. Simon et al. [13] focused on emergency colorectal surgery and showed that frailty is associated with morbidity, mortality, and loss of independence in older patients.

Therefore, previously published data regarding the relationship of frailty with long-term mortality in patients with colorectal cancer should be analyzed with caution.

According to the results obtained, we found that comorbidities prior to intervention and tumor stage are the two strongest predictors of long-term survival in geriatric patients with colorectal cancer. Boakye et al. [14] concluded that comorbidities and frailty are strong predictors of survival in CRC patients but did not adjust for these variables and had a short follow-up duration. The possible mechanisms by which comorbidities might affect the prognosis of patients with colorectal cancer have been well documented by these authors. As we observed in our results, there does not seem to be an association between comorbidities and CRC stage at diagnosis. However, comorbidities might independently increase the risk of non-cancer-related deaths. These patients might also have disabilities and worse post-operative outcomes, which could negatively affect their long-term prognosis. Moreover, these patients are less likely to receive standard cancer treatments such as chemotherapy. Comorbidities may also interact with CRC, affecting tumor biology, accelerating disease progression or increasing the risk of mortality [14].

However, tumor stage at diagnosis is by far the most important factor and is the main consideration with regard to treatment recommendations in CRC care guidelines [42]. In our analysis, the effect of tumor stage on long-term survival was very strong and was comparable to the effect of comorbidities. Age, sex, and other predictive variables, such as nutritional status, were not related to long-term survival in our sample.

Knowledge of these factors in this population may help us appropriately advise the patient and their family during the pre-operative decision-making process. This does not mean that we should simply reject the possibility of surgery in frail patients with comorbidities and advanced cancer stages. A potential modification of the syndrome well in advance of potential surgery may also be included in the benefits of frailty assessment: pharmacological interventions, nutritional supplementation, prehabilitation exercise programmes, etc. [43]. These subjects have not been addressed in this study.

Another important factor to consider here is the quality of life secondary to sustained functional decline, which is common after colon cancer surgery [44]. Reducing the remaining quality of life in these patients would not make sense. This topic was not studied in this report either. Therefore, the decision must be made individually with all the information available on the expected survival and the post-operative quality of life in an attempt to avoid overtreatment or undertreatment, two well-known pitfalls in geriatric oncology [36].

The present study has several limitations. This was a single-center study, and we wondered if a larger sample size would reveal additional variables that were predictive of long-term mortality in the univariate analysis. However, although data were collected prospectively, there was a long follow-up period, and consecutive subject inclusion, in which all of the patients agreed to participate, there may have been a selection bias prior to the referral of each case. It would be interesting to know the median CFS score of patients excluded from surgery before submission to the surgical setting, in comparison to the patients included into the study to shed some light on selection bias, but these data could not be collected. This study also has significant strengths, such as the homogeneity of the sample. All of our patients were treated for colorectal cancer with elective surgery, and the long-term mortality was comparable to that published in other series [45]. A standardized pre-operative geriatric assessment was performed in all the patients in the same pre-operative setting in a truly older population. Therefore, unlike other recently published studies with heterogeneous populations, we consider that the results obtained in this study could be generalized more specifically to the population of older patients with colorectal cancer.

Concerning other factors that could also have influenced the results, it has been suggested that ERAS pathways and minimal invasive surgical technique may play an important role in the successful outcome in older patients after colorectal surgery. In our series, no multimodal rehabilitation protocol was implemented in these old patients. We believe in the benefits of these programs that we are currently applying, but in a recent published trial, Carli et al. [46] concluded that prehabilitation does not seem to improve postoperative outcomes compared with postoperative rehabilitation in frail patients undergoing colorectal cancer resection.

As to laparoscopic surgery, this approach is considered superior to open surgery for frail patients undergoing colon resection. It has been demonstrated that increases in frailty magnify differences between approaches [47]. The rate of intervention performed laparoscopically in our patients was relatively low since during the study period this approach was still being implemented. However, there were no differences in the number of laparoscopic procedures performed between frail and no frail patients, and we found no significant differences in long-term survival depending on the type of approach.

In conclusion, frailty assessed with CSHA-CFS scale is associated with poor short-term post-operative outcomes, but it does not seem to affect long-term survival in patients with colorectal cancer. Instead, high Charlson Comorbidity Index and tumor stage are good predictors of long-term survival. More large-scale studies with adjustment for more prognostic factors are needed. Therefore, frailty should not be considered a contraindication for adequate planning of colorectal cancer treatment in older patients, but it should be individualized taking into account comorbidity and tumor stage rather than frailty itself.

## Data Availability

Not applicable

## Abbreviations

ACS-NSQIP: American College of Surgeons National Surgical Quality Improvement Program
ADL: Activities of daily living
ASA: American Society of Anesthesiology
CCI: Comprehensive Complication Index
CGA: Comprehensive Geriatric Assessment
ChCI: Charlson Comorbidity Index
CI: Confidence interval
CRC: Colorectal cancer
CSHA-CFS: Aging-Clinical Frailty Scale
HR: `Hazard ratio
IQR: Interquartile range
MNA-SF: Mini Nutritional Assessment Short Form questionnaire
SD: Standard deviation
SPMSQ: Short Portable Mental State Questionnaire
